# Evaluating the Correlation of Bariatric Surgery and the Prevalence of Cancers in Obese Patients: A Study of the National Inpatient Sample (NIS) Database

**DOI:** 10.7759/cureus.23976

**Published:** 2022-04-09

**Authors:** Devashish Desai, Sachi Singhal, Jean Koka

**Affiliations:** 1 Internal Medicine, Crozer Keystone Health System, Upland, USA; 2 Internal Medicine, Crozer-Chester Medical Center, Upland, USA; 3 Hematology and Oncology, Crozer-Chester Medical Center, Upland, USA

**Keywords:** bariatric surgery, colorectal cancer, gastric cancer, prostate cancer, renal cell cancer, rcc, breast cancer, esophageal cancer, obesity related cancers, obesity

## Abstract

Purpose

Obesity is a global pandemic that exerts a significant burden on healthcare worldwide. Multiple cancers, as well as deaths from the same, are more prevalent in obese patients. Bariatric surgery has been shown to be the most effective way of treating obesity once other measures have been exhausted. There is no concordant data available to support that bariatric surgery can reduce the prevalence of cancer. Using one of the largest data samples, we evaluate the correlation of bariatric surgery in morbidly obese patients with the prevalence of obesity-related cancers (breast, endometrial, esophageal, colorectal, prostate, and renal) in morbidly obese patients.

Patients and methods

A sample of 7,672,508 morbidly obese patients was identified from the 1994 to 2004 records of the National Inpatient Sample (NIS) database, divided into those who did and did not undergo bariatric surgery, and studied for the prevalence of obesity-associated cancers.

Results

Obesity was predominantly seen in the Caucasian population (68.22%). The mean age of cases who underwent bariatric surgery was younger when compared to those who did not undergo the procedure (43.89±25.16 vs. 54.90±36.40, p-value <0.0001). The highest bariatric surgery rate was seen in the Northeast (5.57%), followed by the West (4.15%), South (3.02%), and Midwest (2.96%) (p-value <0.0001). Overall, the odds of morbidly obese patients who underwent bariatric surgery and developed cancer are: esophageal cancer 0.19 (0.1218-0.3078, p <0.0001), colorectal cancer 0.0368 (0.0275- 0.0493, p <0.0001), endometrial cancer 0.0155 (0.0099-0.0244, p <0.0001), breast cancer 0.0712 (0.0582-0.0871, p <0.0001), prostate cancer 0.0285 (0.0199-0.0408, p <0.0001) and renal cancer 0.0182 (0.0106-0.0314, p <0.0001). The odds of cancer post-bariatric surgery remained significantly lower even after matching certain confounding factors.

Conclusions

The odds of developing breast, esophageal, prostate, renal, and colorectal cancers are significantly lower in morbidly obese patients who undergo bariatric surgery.

## Introduction

Obesity is a health problem that has increased in recent years, with 39% of the adult population worldwide being considered overweight or obese [1]. Multiple cancers are found with higher prevalence in obese patients, the common ones being endometrium, post-menopausal breast, colon, and kidney cancers [2]. Cancer death rates are significantly higher in individuals with a body mass index (BMI) above 40 than in normal-weight individuals (52% higher in men and 62% in women). Around 14% of cancer deaths in men and 20% in women in the USA are estimated in adults over 50 who are overweight or obese [3]. Bariatric surgery is the most effective way for significant weight loss if intentional weight loss is ineffective [4]. Multiple studies have been conducted to evaluate the effects of bariatric surgery in the prevention of obesity-related cancers. However, there is no confirmed concordant data on risk reduction so far. We have utilized one of the largest inpatient sample data in the United States to further evaluate the association in outcomes of bariatric surgery and its effect, if any, on the prevention of various obesity-related malignancies.

## Materials and methods

Methods

Data for this study was obtained from the 1999-2014 Nationwide Inpatient Sample (NIS). The NIS is maintained as part of the Health Care Utilization Project (HCUP) of the Agency for Healthcare Research and Quality (AHRQ). It is the largest all-payer inpatient care database in the US and comprises a 20% stratified random sample of all US hospital discharges. Each discharge in the NIS is de-identified, so all discharges were considered to be independent. The unit for this analysis was each discharge rather than each individual. Further details on the NIS design are available at https://www.hcup-us.ahrq.gov/

Study population

We identified all patients with a primary diagnosis of Morbid Obesity by querying the database using the International Classification of Disease-Clinical Modification, 9th revision (ICD-9 CM) codes "27800" and "27801". The population was divided into two groups, one where patients underwent bariatric surgery and one without bariatric surgery. The population then was studied for the prevalence of different cancers. Most comorbidity, complications, associated symptoms, and procedures were identified by searching all secondary diagnoses fields using the HCUP-defined constellation of ICD-9 codes corresponding to these diagnoses and contained in the HCUP Clinical Classification Software (CCS) as per Table 1. In-hospital mortality and length-of-stay were studied using HCUP variables named "DIED" and "LOS" respectively. The prevalence of cancers was studied in patients with and without bariatric surgery. Comorbidities associated with hospitalization were identified by AHRQ comorbidity measures available in the NIS. AHRQ comorbidity measures identify different comorbidities using ICD-9- CM diagnoses that are likely to have been present prior to hospitalization. The modified Charlson Comorbidity Index (CCI), a validated weighted comorbidities disease severity score composed of 17 comorbid conditions was calculated for all participants. 

**Table 1:** ICD codes used in the study

ICD codes used in the study	
ICD 9 Diagnosis code used	Diagnosis
305.1, 649.0	Smoking
V16.42, V16.51, V16.49, V16.9, V16.0, V16.3	Family History of Cancer
179, 182	Endometrial Cancer
153, 154.0	Colorectal Cancer
150.0	Esophageal Cancer
185	Prostate Cancer
189.0, 189.1	Renal Cancer
ICD 9 Procedures Code used	Procedure
44.68, 44.95, 43.89, 44.31, 44.38, 44.39	Bariatric Surgery

Statistical analysis

We used SAS 9.4 (SAS Institute Inc, Cary, North Carolina) for data analysis. Categorical variables and continuous variables were assessed by the Rao-Scott Chi-square test and the Student's t-test, respectively. A two-tailed alpha of <0.05 was required for statistical significance. Baseline characteristics of participants were summarized using descriptive statistics. We evaluated the prevalence of different cancers in patients with and without a history of bariatric surgery. After the tests on raw data, propensity score matching was performed to reduce selection bias and the effects of confounding variables by balancing the covariates between the two groups. To calculate propensity score, a logistic regression model was fitted with the covariates gender, age, race, hospital teaching status, hospital location, smoking and alcohol consumption history, CCI, and family history of cancer; with bariatric surgery as the case. A 1:3 match was done. The outcome of the matched data was checked for significance using the Rao-Scott Chi-Square Test, and Odds Ratio.

## Results

We identified 7,672,508 (weighted frequency - 37,809,241) cases of morbid obesity over the period of 1999-2014, with 3.6% (n=279,171) having undergone bariatric surgery. Table 2 consists of the demographic details of the study. Females were approximately two-thirds of the cohort. Obesity was predominantly seen in the Caucasian population (68.22%), followed by African Americans (17.89%). 3.81% of Caucasians underwent bariatric surgery, followed by 3.34% of Hispanics and 2.55% of African Americans (p-value <0.0001). The mean age of cases who underwent bariatric surgery was younger when compared to those who did not undergo the procedure (43.89±25.16 vs. 54.90±36.40, p-value <0.0001). A trend analysis of bariatric surgery and the cancer population in the study shows that cancer prevalence is gradually and continuously up trending. In contrast, bariatric surgery utilization has remained almost the same over the years.

**Table 2:** Demographics and insurance status of the cohort, and characteristics of the hospital including location, region and teaching status per admission

Variable		Total (%)	With Bariatric Surgery (% of variable/ % of total)	Without Bariatric Surgery (% of variable/ % of total)	P-Value
Age			43.89±25.16	54.90±36.40	<0.0001
Gender	Males	35.64	2.01 (0.71)	97.99 (34.92)	<0.0001
	Females	64.36	4.52 (2.91)	95.48 (61.45)	
Race	Whites	68.22	3.81 (2.60)	96.19 (65.62)	<0.0001
	Blacks	17.89	2.55 (0.46)	97.45 (17.43)	
	Hispanics	9.97	3.34 (0.33)	96.66 (9.64)	
	Asians and Pacific Islanders	0.9	2.24 (0.02)	97.76 (0.88)	
	Native Americans	0.63	3.00 (0.02)	97.00 (0.61)	
	Others	2.4	4.59 (0.11)	95.41 (2.29)	
Insurance	Medicare	40.07	1.02 (0.41)	98.98 (39.66)	<0.0001
	Medicaid	15.36	1.78 (0.27)	98.22 (15.08)	
	Private	36.05	7.29 (2.63)	92.71 (33.42)	
	Self-pay	4.83	3.78 (0.18)	96.22 (4.64)	
	No charge	0.48	0.77 (0.00)	99.23 (0.48)	
	Other	3.21	4.18 (0.13)	95.82 (3.08)	
Hospital Region	Northeast	16.19	5.57 (0.90)	94.43 (15.29)	<0.0001
	Midwest	24.36	2.96 (0.72)	97.04 (23.64)	
	South	39.98	3.02 (1.21)	96.98 (38.77)	
	West	19.47	4.15 (0.81)	95.85 (18.66)	

In terms of insurance and payment, 40.07% of patients had Medicare, followed by 36.05% on private insurance, and 15.36 had Medicaid. Only 1.02% of Medicare patients underwent bariatric surgery, while 7.29% of patients with private insurance underwent bariatric surgery (p-value <0.0001). Around 4.38% of patients were self-pay, and out of these, 3.78% underwent bariatric surgery. 88.08% of patients were admitted to urban hospitals, and 4.79% of them underwent bariatric surgery, while only 1.52% of patients admitted to rural hospitals underwent bariatric surgery (p-value<0.0001). 39.98% of patients were admitted to hospitals in the southern states, followed by 24.36% in Midwest, 19.47% in the West, and 16.19% in the Northeast (Table 2). The highest bariatric surgery rate was seen in the Northeast (5.57%), followed by the West (4.15%), South (3.02%), and Midwest (2.96%) (P-value <0.0001) regions. Bariatric surgeries were performed more in the teaching facilities even though the patient population seen in these centers was less than that in non-teaching hospitals.

Following is the breakdown of patients with obesity-related cancers in our study: esophageal cancer (N=2,481), colorectal cancer (N=32,310), prostate cancer (27,792), renal cancer (18,882), breast cancer (35,276), and endometrial cancer (32,295). Females still constituted two-thirds of this population. Still, there was no significant difference between bariatric interventions. Caucasians were still the dominant race in patients with these cancers (72.91%), followed by African Americans (15.29%). The utilization of bariatric surgery was mostly seen in Native Americans (0.65%), followed by Asians and Pacific Islanders (0.22%) and Caucasians (0.16%) (P-value = 0.0111). Medicare was the most common insurance provider (47.12%), followed by private insurance (36.05%) and Medicaid (15.36%), with the largest number of bariatric surgery seen in self-payees (0.28%) and private insurance holders (0.20%) (P-value <0.0001) (Table 3).

**Table 3:** Demographic data, insurance status and characteristics of the hospital of admission of our patient cohort with obesity-related cancers, with and without bariatric surgery

Variable		Total (%)	With Bariatric Surgery (%variable/ %total)	Without Bariatric Surgery (%variable/ %total)	P-Value
Gender	Males	36.54	0.13 (0.05)	99.87 (36.49)	0.1901
	Females	63.46	0.16 (0.10)	99.84 (63.36)	
Race	Whites	72.91	0.16 (0.12)	99.84 (72.79)	0.0111
	Blacks	15.29	0.14 (0.02)	99.86 (15.26)	
	Hispanics	7.97	0.08 (0.01)	99.92 (7.97)	
	Asians and Pacific Islanders	1.07	0.22 (0.00)	99.78 (1.07)	
	Native Americans	0.49	0.65 (0.00)	99.35 (0.48)	
	Others	2.27	0.10 (0.00)	99.90 (2.27)	
Insurance	Medicare	47.12	0.10 (0.05)	99.90 (47.08)	<0.0001
	Medicaid	8.2	0.13 (0.01)	99.87 (8.19)	
	Private	39.94	0.20 (0.08)	99.80 (39.86))	
	Self pay	2.16	0.28 (0.01)	99.72 (2.15)	
	No charge	0.32	0.00 (0.00)	100.00 (0.32)	
	Other	2.25	0.09 (0.00)	99.91 (2.25)	
Hospital Region	Northeast	16.52	0.20 (0.03)	99.80 (16.480	0.0613
	Midwest	24.95	0.12 (0.03)	99.88 (24.92)	
	South	36.18	0.13 (0.05)	99.87 (36.14)	
	West	22.35	0.16 (0.04)	99.84 (22.31)	
Hospital Teaching Status	Teaching	52.78	5.38 (2.34)	94.62 (41.26)	<0.0001
	Non Teaching	56.39	3.65 (2.06)	96.35 (54.34)	

As per Table 4, it is seen that the odds of having cancer were significantly lower in obese patients who underwent bariatric surgery. This stands true for all the cancer types in our study. On matching some confounding factors (such as gender, age, hospital location, academic status, race, history of alcohol abuse and smoking, family history of cancer, and Charlson index), it was seen that odds of having cancer were still significantly lower in obese patients who underwent bariatric surgery compared to who did not (seen in Table 5).

**Table 4:** Prevalence of obesity-related cancers by cancer type, with and without bariatric surgery in the cohort

Variable		Total (N(%))	With Bariatric Surgery (N(%))	Without Bariatric Surgery (N(%))	OR (95% CI)	P-Value
Esophageal Cancer	Present	2,481 (0.03)	18 (0.73)	2,463 (99.27)	0.1936 (0.1218 - 0.3078)	<0.0001
	Absent	7,674,783 (99.97)	279,153 (3.64)	7,395,630 (96.36)		
Colorectal Cancer	Present	32,310 (0.42)	45 (0.14)	32,265 (99.86)	0.0368 (0.0275 - 0.0493)	<0.0001
	Absent	7,644,954 (99.58)	279,126 (3.65)	7,365,828 (96.35)		
Endometrial Cancer	Present	32,295 (0.42)	19 (0.06)	32,276 (99.94)	0.0155 (0.0099 - 0.0244)	<0.0001
	Absent	7,644,969 (99.58)	279,152 (3.65)	7,365,817 (96.35)		
Breast Cancer	Present	35,276 (0.46)	95 (0.27)	35,181 (99.73)	0.0712 (0.0582 - 0.0871)	<0.0001
	Absent	7,641,988 (99.54)	279,076 (3.65)	7,362,912 (96.35)		
Prostate Cancer	Present	27,792 (0.36)	30 (0.11)	27,762 (99.89)	0.0285 (0.0199 - 0.0408)	<0.0001
	Absent	7,649,472 (99.64)	279,141 (3.65)	7,370,331 (96.35)		
Renal Cancer	Present	18,882 (0.25)	13 (0.07)	18,869 (99.93)	0.0182 (0.0106 - 0.0314)	<0.0001
	Absent	7,658,382 (99.75)	279,158 (3.65)	7,379,224 (96.35)		

**Table 5:** Prevalence of obesity-related cancers with and without bariatric surgery after propensity matching

Variable		Total (N(%))	With Bariatric Surgery (N(%))	Without Bariatric Surgery (N(%))	OR (95% CI)	p-Value
Esophageal Cancer	Present	88 (0.0126)	10 (11.3636)	78 (88.6364)	0.3846 (0.1988 - 0.7439)	0.0031
	Absent	699,052 (99.9874)	174,775 (25.0017)	524,277 (74.9983)		
Colorectal Cancer	Present	1044 (0.1493)	22 (2.1073)	1,022 (97.8927)	0.0645 (0.0422 - 0.0985)	<0.0001
	Absent	698,096 (99.8507)	174,763 (25.0342)	523,333 (74.9658)		
Endometrial Cancer	Present	2966 (0.4242)	10 (0.3372)	174,775 (25.1051)	0.0101 (0.0054 - 0.0187)	<0.0001
	Absent	696,174 (99.5758)	2,956 (99.6628)	521,399 (74.8949)		
Breast Cancer	Present	712 (0.1018)	55 (7.7247)	657 (92.2753)	0.2509 (0.1875 - 0.3357)	<0.0001
	Absent	698,428 (99.8982)	174,730 (25.0176)	523,698 (74.9824)		
Prostate Cancer	Present	663 (0.0948)	21 (3.1674)	642 (96.8326)	0.0980 (0.0637 - 0.1509)	<0.0001
	Absent	698,477 (99.9052)	174,764 (25.0207)	523,713 (74.9793)		
Renal Cancer	Present	999 (0.1429)	7 (0.7007)	992 (99.2993)	0.0211 (0.0101 - 0.0443)	<0.0001
	Absent	698,141 (99.8571)	174,778 (25.0348)	523,363 (74.9652)		

## Discussion

Obesity is a global pandemic, reaching numbers as high as 60-70% of the entire adult population in some world regions. The burden of cancer attributable to obesity is 11.9% in men and 13.1% in women (expressed as population attributable fraction) [1-2]. It has been noted to be the second most prevalent preventable cause of cancer after tobacco use [3]. Lifestyle modifications to reduce weight show significant changes in the expression of cancer-associated biomarkers, including but not limited to estrogens, TNF-alpha, IL-6, and other inflammatory markers [4,5]. Other than intentional weight loss, bariatric surgery is efficacious in achieving this goal in morbidly obese patients who have failed these lifestyle changes [6]. Bariatric surgery has shown a significant reduction of cardiovascular, endocrine, infectious, psychiatric, and infectious risk in morbidly obese patients in the past with a high reduction in risk of death as well [7]. Despite its largely positive impact on this subset of the population, it appeared to be largely underutilized, with only 3.6% of morbidly obese patients in our cohort undergoing this readily available procedure. One of the reasons may be insurance coverage: most number of patients undergoing bariatric surgeries paid out of pocket (Tables 2-3). Another plausible reason is the cost burden. Gasoyan et al. explore this premise in their research and compare long-term expenditures in treating extreme obesity and associated comorbidities with bariatric surgery versus conservative or traditional therapies. They find no significant difference in cost overall. Bariatric surgery as an option may not appear as cost savings to the population but has been found to be cost-effective [8]. Other possible reasons for underutilization may be a lack of referrals from primary care physicians for patients who qualify for bariatric surgeries [9].

According to a few meta-analyses, there is a reduction in obesity-related cancer, including colorectal, esophageal, endometrial, prostate, and breast cancers. There is no definite conclusion on bariatric surgery advantages for breast cancers, as some studies showed benefits of bariatric surgery in the reduction of breast cancers, some said only certain types of breast cancer could be reduced, and some studies did not show any benefit whatsoever [6,10]. A multisite prospective study showed a substantial risk reduction in developing several cancers in severely obese people who underwent bariatric surgery compared to a similar population who didn’t proceed with the surgery. Declines in cancer risk were seen most strongly in postmenopausal breast cancer, endometrial cancers, and colorectal cancer, concurrent with our findings. The odds of having cancers were significantly lower in obese patients who underwent bariatric intervention [11]. A retrospective study further shed light on the risk of developing uterine malignancy in females across the United States, registered in the US University Health System Consortium - the RR was 0.19 for women who had undergone bariatric surgery, and even lower (0.17) for the women who underwent surgery and maintained a normal weight [12]. 

The relationship between GERD and increasing weight is strong and linear [13]. The Norwegian HUNT study displayed a reduction of symptoms and increased treatment success with weight loss in a dose-dependent fashion [14]. Barrett’s esophagus was also significantly more prevalent with weight gain (35% increase with a 5-point increase in BMI) [15]. Both GERD and Barrett’s esophagus are the most substantial known risk factors for esophageal adenocarcinoma, and its rise in prevalence has paralleled the increase in obesity [16]. On the flip side, one of the significant drawbacks of bariatric surgery is the possibility of inducing or worsening GERD, especially with sleeve gastrectomy. There are some reports of EAC development after bariatric surgery, and with the stomach no longer available to create a path, more complicated surgeries have to be undertaken [17]. In our study, the odds of having esophageal cancer were significantly lower in obese patients who underwent bariatric surgery.

Obesity is also a well-established risk factor for renal cell cancer (RCC). A strong correlation is seen in both females and males and is even stronger when BMI is elevated at age 50 [18]. The mechanism is unclear but, in both prostate and renal cancers and insulin resistance, is associated with high circulating levels of insulin-like growth factors (ILGF) and high levels of the hormone leptin - which activates several carcinogenic cellular pathways. In a more recent retrospective case-control analysis by Wang et al., higher C-peptide, IL-6, and TNF-a levels had a much higher correlation with RCC development, further supporting this association [19]. Although an abundance of data exists supporting this premise, there are some studies that have reported different outcomes. Srikanth et al. report their 11-year experience with RCC following bariatric surgery, discussing five patients with mean BMIs of 26 with incidental RCCs 8-66 months post-operation [20]. Further studies need to be undertaken to explore whether these are truly incidental, or if there is an increased lifetime risk of developing RCC in once-obese patients.

A modest yet consistent association is established between prostate cancer and obesity; however, it was found to be much more significant in Europe and Australia on stratifying the population. In the USA, PSA screening is widespread, and importantly, obesity is related to falsely reduced PSA levels, thereby complicating the situation [21]. Despite these regional differences, extensive studies have consistently demonstrated a dose-response relationship between obesity and fatal prostate cancer [22]. Whether reversing obesity slows cancer growth-this remains unknown.

Our analysis noted that females constitute two-thirds of the population and have undergone bariatric surgery more than males. Also, Caucasians were predominantly the race to have obesity and undergo bariatric surgery. Even though African Americans constituted the second most common race, the incidence of them undergoing bariatric surgery was lesser than Hispanics. Medicare was the largest insurance provider, though the utilization of bariatric procedures was the least when compared to other insurances. The cancer rates were also higher in patients with Medicare when compared to other insurance providers. The patients in the Northeastern region underwent the most reported bariatric surgeries compared to other regions, even though their obese population was the least in number overall. Increasing awareness regarding the perils of obesity in the areas that are dense in terms of this patient population is necessary, and bariatric surgeries should be offered more frequently than noted in our study. It is also imperative that Medicare helps patients monetarily get bariatric surgeries more than in the present-day scenario.

An abundance of data supports obesity being one of the most significant preventable driving forces behind carcinogenesis. Both preventing and treating morbid obesity results in risk reduction of obesity-associated cancers. While we do not recommend bariatric surgery to manage elevated cancer risk and its reduction, it has an added beneficial effect on these cancers risk reduction while targeting morbid obesity that has failed all other interventions.

Limitations

This study has limitations associated with administrative claims data, which contains codes produced for billing and documentation purposes. Being a retrospective observational study, we can only report an association between patients undergoing bariatric surgery and the prevalence of cancer. Our analysis was limited to strictly in-patient admissions, and our study lacks data from clinics. An in-depth study is required to explore the relationship between controlling and managing the comorbidities along with the surgery and its effect on the reduction of the prevalence. A prospective cohort study studying patients with morbid obesity over the years would be required to clearly define the trends in forthcoming years. Since ICD-9-CM codes were used to identify all the diagnoses and associated comorbid conditions, the possibility of coding errors cannot be overlooked. Also, our study, being strictly NIS-based, cannot comment on the degree of weight loss achieved by the patients. It is possible that after undergoing bariatric surgery, obesity was removed from the problem list, hence resulting in a lower number of cases of bariatric surgery.

## Conclusions

With the increasing prevalence of obesity and obesity-related cancers, it is imperative to evaluate the preventive measures to reduce both. Bariatric surgery has been reported to be the most efficient way of reducing weight after failing intentional weight loss measures and needs to be further studied in terms of its effects on obesity-related cancers. Our study identifies significantly decreased odds of developing the reported malignancies in patients who are obese and underwent bariatric surgery. The study does not claim that bariatric surgery should be used for the prevention of cancers but rather highlights the fact that weight loss is the key to decreasing cancer risk. To verify our findings, a prospective cohort needs to be undertaken. With advancements in the procedure techniques and reduction of side effects, bariatric surgery, though not currently approved and not advised, can be offered as the next step in these morbidly obese patients for prevention and risk reduction of obesity-related cancers.
